# Functional Comparison between VP64-dCas9-VP64 and dCas9-VP192 CRISPR Activators in Human Embryonic Kidney Cells

**DOI:** 10.3390/ijms22010397

**Published:** 2021-01-01

**Authors:** Nasir Javaid, Thuong L. H. Pham, Sangdun Choi

**Affiliations:** 1 Department of Molecular Science and Technology, Ajou University, Suwon 16499, Korea; nasirjavaid@ajou.ac.kr (N.J.); plhthuong@gmail.com (T.L.H.P.); 2 S&K Therapeutics, Woncheon Hall 135, Ajou University, Suwon 16499, Korea

**Keywords:** CRISPR/dCas9, gene activation, reprogramming factor, CRISPR activator

## Abstract

Reversal in the transcriptional status of desired genes has been exploited for multiple research, therapeutic, and biotechnological purposes. CRISPR/dCas9-based activators can activate transcriptionally silenced genes after being guided by gene-specific gRNA(s). Here, we performed a functional comparison between two such activators, VP64-dCas9-VP64 and dCas9-VP192, in human embryonic kidney cells by the concomitant targeting of *POU5F1* and *SOX2*. We found 22- and 6-fold upregulations in the mRNA level of *POU5F1* by dCas9-VP192 and VP64-dCas9-VP64, respectively. Likewise, *SOX2* was up-regulated 4- and 2-fold using dCas9-VP192 and VP64dCas9VP64, respectively. For the POU5F1 protein level, we observed 3.7- and 2.2-fold increases with dCas9-VP192 and VP64-dCas9-VP64, respectively. Similarly, the SOX2 expression was 2.4- and 2-fold higher with dCas9-VP192 and VP64-dCas9-VP64, respectively. We also confirmed that activation only happened upon co-transfecting an activator plasmid with multiplex gRNA plasmid with a high specificity to the reference genes. Our data revealed that dCas9-VP192 is more efficient than VP64-dCas9-VP64 for activating reference genes.

## 1. Introduction

The nuclear compartmentalization of DNA in eukaryotic cells is achieved by its association with histone proteins to form a structure called chromatin whose fundamental unit is the nucleosome. The folding pattern has categorized chromatin into euchromatin and heterochromatin, which are marked by multiple modifications such as acetylation, methylation, phosphorylation, ubiquitination, sumoylation, etc. [1,2,3]. The transcriptionally less active part of the DNA constitutes tightly folded chromatin, known as heterochromatin [4,5]. The degree of chromatin folding and associated gene expression is controlled in various biological processes such as transcription, recombination, and replication [6,7,8]. Hence, the transcriptional activation of epigenetically silenced genes is required for various commercial and therapeutic applications. For example, the reprogramming of specialized cells into induced pluripotent stem cells (iPSCs) by activating endogenous genes has been achieved using gene activators [9,10]. Various transcription factors bind to the DNA sequence of promoter and enhancer regions of target genes and use their effector domains to change the associated gene expression [11]. Artificial transcription factors (ATFs) have been developed based upon the same principle by binding the DNA binding domain with a functional domain to activate the expression of target genes [12,13]. The DNA specificity of transcription activator-like effectors (TALE) and zinc finger proteins has been explored to bind and activate the expression of target genes [14,15,16,17,18,19,20]. However, these ATFs are less favorable because of their lower specificity, time-consuming nature, and complex design.

The clustered regularly interspaced short palindromic repeats (CRISPR)/CRISPR-associated protein (Cas) system has been gaining popularity as a powerful genome-editing tool. This system is classified into multiple types (type I-VI) based upon the signature proteins and working mechanism [21]. Out of them, the type II system is composed of Cas9 protein, CRISPR RNA (crRNA), and trans-activating cRNA (tracrRNA) [22]. The Cas9 protein cleaves the dsRNA at a site 3 bp upstream to the protospacer adjacent motif (PAM) with the help of two DNA cleavage domains HNH and RuvC [23]. The cleaved target DNA is repaired by host DNA repair pathways—i.e., nonhomologous end joining (NHEJ) or homology directed repair (HDR) [23]. This genome editing ability of the CRISPR/Cas system has made it a potential tool for various applications. For example, it is being used to study or treat numerous cancers [24,25,26]; autoimmune, inflammatory, and immunodeficient conditions [27,28,29,30]; as well as viral [31,32,33,34,35], vector-transmitted [27,36,37,38], and genetic diseases [39,40,41,42]. Moreover, it is widely being used in the agricultural industry to genetically modify various crops such as rice, wheat, maize, cotton, tobacco, etc. [43,44,45,46,47]. Upon introducing the Cas9 system into infected cells, the short guide RNAs (gRNAs)—which have been pre-designed to target specific viral genomic elements—help to inactivate or eliminate the viral load from the infected cell [33]. In addition, some disease-related mutations in animals have been cured using the Cas9-mediated genome-editing system [48,49]. Furthermore, Cas9 can alter multiple genomic loci through the simultaneous introduction of multiple gRNAs into the cell [50,51].

One of the genetically modified versions of Cas9 nuclease with promising applications such as generating iPSCs is named “dead Cas9” or “deactivated Cas9” (dCas9) [9,10,52]. The dCas9 was made by introducing a D10A point mutation into the RuvC nuclease domain and an H840A point mutation into the HNH nuclease domain of the wild-type Cas9 enzyme [53,54]. The dCas9 has the ability to bind target DNA sequences similar to those of the wild-type Cas9; however, it cannot cleave DNA owing to the loss of its endonuclease cleavage activity [55]. Hence, dCas9 has been linked to various regulatory domains, such as an activator or repressor, to control diverse biological processes [9,56]. The linked-dCas9 is guided by the gRNA to identify the target sequence adjacent to the protospacer adjacent motif (PAM) by avoiding non-specific binding [57]. The CRISPR/dCas9 activator system was made by fusing it to the transcription activator domain(s), which recruits RNA polymerase around the binding site to activate the transcriptionally silenced gene [58,59].

The VP16-based CRISPR/dCas9 activators have been widely used and consist of multiple repeats of the minimal activation domain of VP16 protein (unit sequence: PADALDDFDLDML) derived from Herpes simplex virus [50,60,61]. The CRISPR/dCas9 activators with a various number of repeat units and their orientations have been reported, such as dCas9-VP64 [62], VP64-dCas9-VP64 [63,64], dCas9-VP64-Rta [65], and dCas9-VP192 [9], etc. These activator systems are being used for various purposes, such as activating inherited disease-causing genes [66] and cellular reprograming and differentiation [9,63,64]. The dCas9-VP64 has been tested to activate the reprogramming gene, *POU5F1* (also named as *OCT4*), by using a single gRNA or multiple gRNAs in combination [67]. The dCas9-SunTag-VP64 activates the *POU5F1* and *SOX2* genes when guided by a single gRNA or a pool of gRNAs targeting each of the genes [10]. Similarly, VP64-dCas9-VP64 has been used to directly reprogram fibroblasts into myocytes [64] and neuronal cells [63] by using a single gRNA or co-transfecting multiple gRNAs. To get the synergistic effect of multiple gRNAs, their transfection or transduction as separate entities might vary the individual number of successfully taken molecules, and a single cell might not have all the required gRNAs; hence, the expression of multiple gRNAs from a single plasmid is preferable [68,69,70]. The multiplex assembly of *POU5F1*-targeting gRNAs was reported to activate the endogenous expression with dCas9-VP192 [9]. Nonetheless, the limited activation of target genes might hinder achieving the targeted applications with a full efficiency [71]. Therefore, a highly efficient activator in combination with a multiplex assembly of gene-targeting gRNAs is needed to increase the expression level of a target gene.

The degree of activated expression depends upon various factors, of which “type of CRISPR activator” is under consideration in our study. In this study, we used a single multiplex plasmid containing gRNAs targeting both *POU5F1* and *SOX2* genes simultaneously in order to perform a comparative functional analysis of two CRISPR activators, VP64-dCas9-VP64 and dCas9-VP192, at the mRNA and protein level. We used human embryonic kidney (HEK293T) cells because of their wide applications in molecular biology. Although these two activators have been reported previously, their mutual functional comparison with these reference genes in the same experimental conditions has not been completed yet. Concluding a better activator by comparative study will be an improvement in CRISPRa in terms of selecting a more efficient factor to activate the expression of the target gene of interest.

## 2. Results

### 2.1. dCas9-VP192 Activator Is More Efficient Than VP64-dCas9-VP64 to Induce mRNA Expression

**Figure 1:**
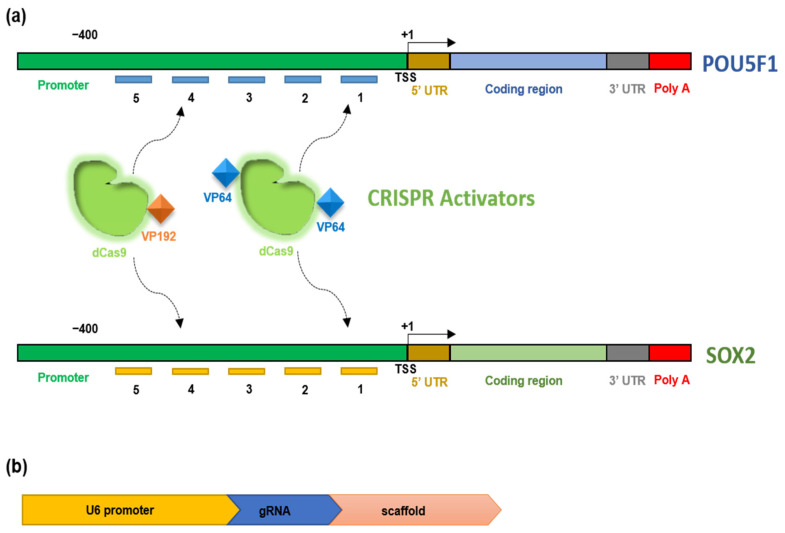
Construction of multiplex plasmid targeting *POU5F1* and *SOX2* (**a**) Graphical depiction of the promoter region of *POU5F1* and *SOX2* genes, where number 1–5 represents the particular gRNA. (**b**) The expression cassette in each array plasmid constitutes U6 promoter, cloned gRNA, and scaffold. TSS, transcription start site. UTR, untranslated region. dCas9, deactivated CRISPR-associated protein 9. VP, virus protein. gRNA, guide RNA.

We used Zhang Lab’s web tool (http://crispr.mit.edu/, Cambridge, MA, USA) to design five gRNAs for both reference genes, *POU5F1* and *SOX2*, targeting almost 400 bp upstream to the transcription start site (TSS) [72]. The graphical depiction of the target region and expression cassette in an array plasmid is shown in Figure 1. For the simultaneous expression of these gRNAs from a single plasmid, we first cloned each gRNA into a separate array plasmid, followed by cloning all the array plasmids into a single multiplex plasmid, which was confirmed by colony PCR and sanger sequencing (Figure S1). The graphical depiction of the cloning strategy used in our study is shown in Figure S1a.

**Figure 2:**
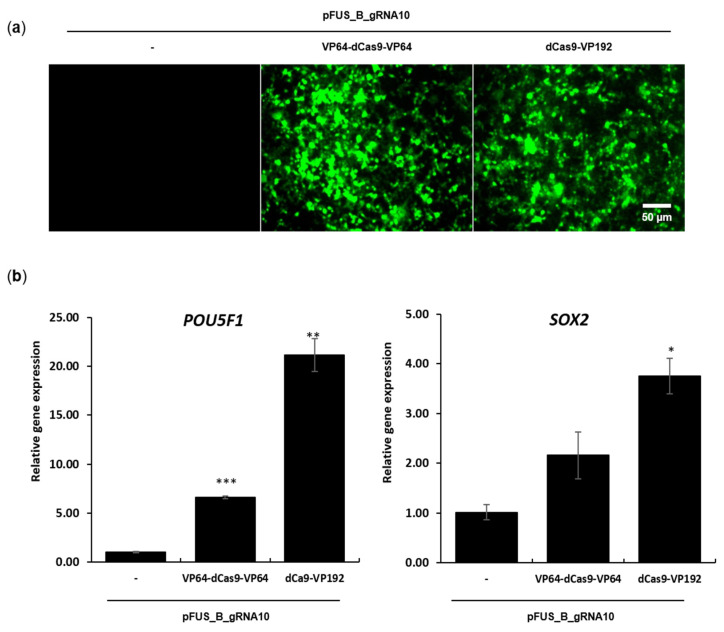
Activation of mRNA expression by CRISPR activators. (**a**) pFUS_B_gRNA10 was transfected alone and with VP64-dCas9-VP64 or dCas9-VP192 plasmid separately at the molar ratio of 1:1 by means of Lipofectamine 3000. The transfected cells were visualized under an inverted microscope (OLYMPUS IX53) after 24 h of transfection. Only the pFUS_B_gRNA10 transfected cells served as a negative control. (**b**) After 72 h of transfection, total RNA was isolated and proceeded for a gene expression analysis of *POU5F1* and *SOX2* via the SYBR Green detection method. CyclophilinG gene served as an endogenous control, while the control represents only pFUS_B_gRNA10 transfected cells. The primers specific to each gene are enlisted in Table S2. The represented values are the average of three independent experiments, where bars represent ± SEM (* *p* < 0.05, ** *p* < 0.01, *** *p* < 0.001) according to Tukey’s method and one-way ANOVA. Scale bar, 50 μm.

The multiplex expression plasmid (pFUS_B_gRNA10) was transfected alone as well as in combination with either VP64-dCas9-VP64 or dCas9-VP192 into human HEK293T cells in a 1:1 molar ratio. After 24 h of transfection, a GFP signal (a gene cloned in each activator plasmid) was detected with a fluorescence microscope. Both of the co-transfected combinations markedly expressed a GFP signal, indicating successful transfection (Figure 2a).

After 72 h of transfection, the total RNA was isolated, reverse-transcribed into cDNA, and used for RT-PCR. Both activator plasmids increased the mRNA expression of *POU5F1* and *SOX2* as compared to the control (only pFUS_B_gRNA10) (Figure 2b). In the case of *POU5F1*, we found a 22-fold and 6-fold upregulation by dCas9-VP192 and VP64-dCas9-VP64, respectively. Likewise, *SOX2* was up-regulated 4-fold and 2-fold by dCas9-VP192 and VP64-dCas9-VP64, respectively. By relative comparison, the activation level of *POU5F1* mRNA by the dCas9-VP192 activator was 3.7-fold (=22/6) greater than that of VP64-dCas9-VP64. Similarly, the *SOX2* activation by the dCas9-VP192 activator was 2-fold (=4/2) greater than that of VP64-dCas9-VP64 (Figure 2b).

### 2.2. dCas9-VP192 Activator Is Also Efficient at Protein Level Than VP64-dCas9-VP64

**Figure 3:**
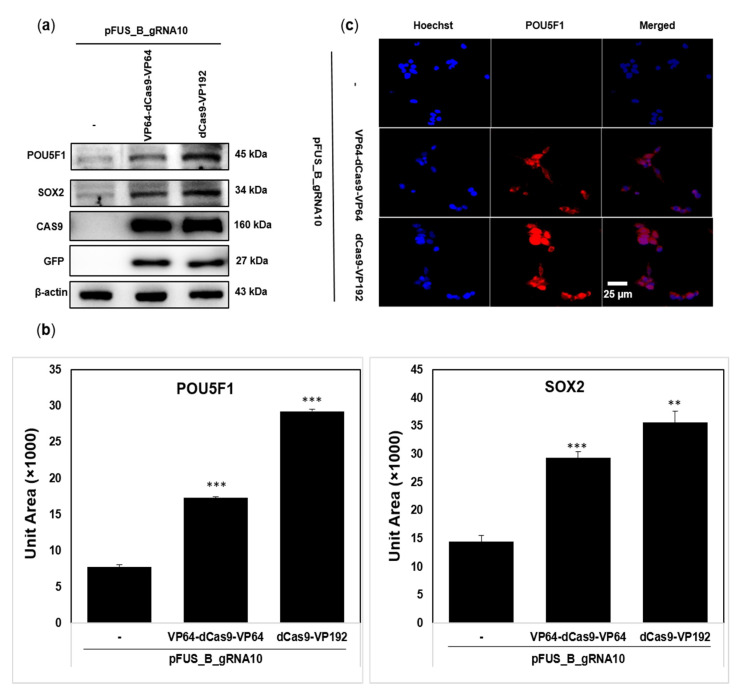
Regulation of the POU5F1 and SOX2 protein expression by CRISPR activators. (**a**) After 72 h of transfection with pFUS_B_gRNA10 alone or in combination with VP64-dCas9-VP64 or dCas9-VP192, the total cell proteins were isolated, separated, transferred, and immunoblotted with the relevant antibodies, as shown in the image. β-Actin served as a loading control. (**b**) Quantification of POU5F1 and SOX2 was conducted on the basis of their band intensities in the Western blot by using the ImageJ software. The absolute quantification values are the means of three independent measurements of their respective band intensities, represented in the form of unit area. The statistical comparison was carried out between cotransfected and single-plasmid-transfected values, where bars represent ± SEM (** *p* < 0.01, *** *p* < 0.001) by Tukey’s method and one-way ANOVA. (**c**) The transfected cells were subjected to immunostaining and fluorescence microscopy after 72 h of transfection. The images were captured with an inverted microscope (Olympus IX53), where a blue color corresponds to nuclei stained with Hoechst 33,258 while the red color represents the POU5F1 protein. Scale bar, 25 μm.

Based on the expressional changes in mRNA, we hypothesized the presence of a similar pattern at the protein level. To confirm this, we transfected HEK293T cells for 72 h with the plasmid combinations as explained above and analyzed the extracted proteins through Western blotting using the primary antibodies specific to POU5F1 and SOX2. We observed the activation of POU5F1 and SOX2 proteins with both activator plasmids as compared to the control (only pFUS_B_gRNA10 transfected cells) (Figure 3a). The densities of the protein bands on each blot were used to construct histograms of protein expression by using the ImageJ software (Figure 3b). A 3.7-fold and 2.2-fold increase for POU5F1 was observed with dCas9-VP192 and VP64-dCas9-VP64, respectively. Similarly, the SOX2 expression was 2.4-fold and 2-fold higher with dCas9-VP192 and VP64-dCas9-VP64, respectively (Figure 3b). However, we did not observe any change in the expression level of CAS9 as well as GFP protein by both activators—i.e., dCas9-VP192 and VP64-dCas9-VP64 (Figure 3a). This indicates that the higher efficiency of dCas9-VP192 as compared to VP64-dCas9-VP64 is not because of the difference in the expression level of dCAS9 protein between them but due to their own attributes.

Despite its specificity, the CRISPR/Cas9 system has been reported to have some off-target effects (30–34). In order to address these, we performed immunoblotting by randomly picking some off-targets—for instance, nuclear factor-kappa B (NF-κB—p65 subunit) and mitogen-activated protein kinases (MAPKs such as JNK and ERK). As expected, we did not observe activation of any of these targets by both dCas9-VP192 and VP64-dCas9-VP64 activators (Figure S2). We also confirmed that the effect seen in our results is the combined response by designed gRNAs and CRISPR activators, but not by only transfecting reagent, only plasmid, only gRNAs, or only activators, etc. This was achieved by performing experiments with various controls, including (1) control (only cells), (2) irrelevant plasmids (pEGFP-N1), (3) only lipofactamine3000 (lipo3000), (4) lipo3000 + dCas9-VP192, (5) lipo3000 + VP64-dCas9-VP64, (6) lipo3000 + pFUS_B_gRNA10, (7) lipo3000 + pFUS_B_gRNA10 + VP64-dCas9-VP64, (8) lipo3000 + pFUS_B_gRNA10 + dCas9-VP192, and (9) lipo3000 + pFUS_B_gRNA10 + pEGFP-N1. After 72 h of transfection, we extracted the whole protein for Western blotting with antibodies specific to POU5F1 and SOX2 and found activation with only combination 7 and 8 but not with others (Figure S2). We also predicted the off-targets for our designed gRNAs using the Cas-OFFinder computational tool (Data File S1 (Supplementary Materials)) and evaluated the expression level of the genes with off-targets located between 0 and −400 bp from TSS. We did not find any significant change in their expression level by our designed gRNAs (Figure S3).

Because of the marked difference in the expression of POU5F1 by two activators, we further confirmed this by the immunostaining of the 72 h post-transfected cells. Both activators with multiplex plasmid robustly activated the expression of POU5F1 protein (Figure 3c). Nevertheless, there was a slight difference in the fluorescence intensity with each activator plasmid.

## 3. Discussion

The advanced sequencing technologies have categorized the transcribed loci based upon the instructions carried by them [73]. It is challenging to comprehend the biological functions of these transcripts and their effects on development and diseases. The function of many genes is still unknown despite advancements in molecular biology. The biological roles of various noncanonical transcripts such as lncRNAs, antisense RNAs, enhancer RNAs, or intergenic RNAs, are still not clear [74]. These secrets could be understood by adopting the molecular techniques able to control the expression of individual transcripts.

RNA interference (RNAi) has been used for a long time to find the function of target genes by disrupting their expression [75]; however, this technique also shows some off-targets [76,77,78]. As an alternative approach, sequence-specific endonucleases such as zinc finger nucleases, transcription activator-like effector nucleases (TALENs), and CRISPR/Cas9 are used [79,80]. Because of its simplicity, efficacy, and specificity, the CRISPR/Cas9 system has gained significant attention for various applications, such as loss-of-function screens, point-mutations, and transcription silencing/activation [50,80,81,82,83,84,85]. The control of gene expression using synthetic transcription factors has multiple scientific and medical applications, such as tissue regeneration [86], the activation of tumor suppressors [87], stem cell differentiation [88,89], and performing genetic screening [90,91]. The deactivated version of Cas9 (dCas9) has been exploited for the transcriptional silencing or activation of target gene(s) [50,92,93]. The fusion of an effector domain to dCas9 has been reported to activate the transcription of reporter genes in human cells and Escherichia coli [50,92].

A single gRNA is able to guide the activator system for activating the transcription of the target gene; however, the effect was more prominent when multiple gRNAs were used in synergy [19,20,55,93]. So, we designed five gRNAs targeting up to 400 bp upstream of the transcription start site of each gene, as targeting this region could activate their transcription [72]. Instead of using individual gRNAs separately, we used them all together in a single transfection by developing a multiplex expression plasmid (Figure 1) to avoid a variable transfection efficiency among individual gRNAs and cell toxicity because of multiple transfections [94,95,96,97,98].

The first-generation CRISPR-based activators simply used dCas9-VP64 for the transcriptional activation of the target gene(s) [50]. The further development of CRISPR activators suggested that the dCas9-VP64 version is less effective than other advanced versions—for instance, VP64-dCas9-VP64 [64] and dCas-VP192 [9]. The former has an equal number of repeat units of the transactivation domain at both ends of dCas9, while the latter one has a greater number of repeat units at the single end of dCas9. Here, we compared their individual efficiency in human embryonic kidney cells (HEK293T) by targeting *POU5F1* and *SOX2* as reference genes. The purpose of developing both end-tagged CRISPR activators, VP64-dCas9-VP64, was to recruit the transcriptional machinery more effectively [64,99]. Nonetheless, according to our results both termini tagged dCas9 as less effective than the one tagged at one termini with a relatively greater number of repeats. We found that the dCas9-VP192-containing activator plasmid is more effective at both the RNA and protein level to activate the *POU5F1* and *SOX2* genes in HEK293T cells (Figure 2 and Figure 3). This could be because of the presence of more VP16 units in dCas9-VP192-containing plasmids. Our results are supported by some previous reports which explain that the extent of the transcriptional activation of endogenous genes is affected by the number of VP16 repeated units [9,63]. This indicates that the number along with the orientation of activation domains should be emphasized when designing new activators in the future. However, adding too many VP16 repeat units could also affect the expression and stability of dCas9, so the selection of a suitable number is important [100]. The DNA binding and cleavage activity of Cas9 involve various conformational changes [101,102,103]; hence, it is possible that the stronger effect by dCas9-VP192 is because of its more favorable conformation rather than the number of VP16 units itself. There is a need to study the conformational changes occurring in CRISPR activator systems during their function. We also observed that reference targets were differently activated by VP64-dCas9-VP64 or dCas9-VP192 at both the mRNA and protein level, which has also been reported previously with other activator systems [59,104]. This differential activation of reference genes could be because of gRNA target sites, epigenetic status, or the basal expression level of the target genes [59,104,105,106]. Besides the *POU5F1* and *SOX2* genes, examining the effect of these activators on additional genes (expressed at an average or higher level) will further support the current findings. Second-generation CRISPR activators are more efficient than original first-generation dCas-VP64 [59]. Based on our results, the activity of second-generation activators could be further improved by optimally increasing the repeat units of transactivation domain VP16.

Human iPSCs are promising for in vitro disease modelling and regenerative medicine because of their close resemblance to embryonic stem (ES) cells in their pluripotency, gene expression, and epigenetic states. The reprogramming of somatic cells into iPSCs can be carried out by injecting its nucleus into an oocyte [107,108,109,110] or by fusing it with an embryonic stem cell [111,112]. The Yamanaka team identified the key transcription factors whose overexpression enabled the reprogramming of somatic cells into iPSCs [113]. Afterwards, the generation of iPSCs became possible from mouse and human somatic cells with the ectopic expression of reprogramming factors such as *POU5F1*, *SOX2*, *KLF4*, *c-MYC*, *NANOG*, and/or *LIN28* [114,115,116,117,118,119,120]. The utility of iPSCs is limited because of two issues: (i) their low efficiency and (ii) the integration of viral transgenes, especially oncogenes such as *KLF4* and *c-MYC*. The reactivation of *c-MYC* is reported to cause tumors in iPSCS-driven chimera mice [114,121]. The iPSCs generated by *POU5F1*, *SOX2*, and *KLF4* showed a low efficiency [121,122]. It is reported that only *POU5F1* and *SOX2* can reprogram primary human fibroblasts by combining histone deacetylase inhibitor, valporic acid [123]. The iPSCs generation has also been reported by using a single gene, *POU5F1*, in combination with some small molecules [124,125]. The reference genes targeted in our study, *POU5F1* and *SOX2*, have the potential to reprogram specialized cells into iPSCs [9,10], which could be used in drug discovery, disease modelling, and regenerative medicine [126,127]. Therefore, the usage of a comparatively more efficient transcriptional activator will facilitate the efficient generation of iPSCs for research and therapeutic purposes.

## 4. Materials and Methods

### 4.1. Cell Line and Culturing Condition

HEK293T (ATCC^®^ CRL-3216™) cells were cultured in high-glucose Dulbecco’s modified Eagle’s medium (DMEM) supplemented with a 1% solution of penicillin (100 U/mL) and streptomycin (100 μg/mL) and 10% fetal bovine serum (FBS) (Thermo Fisher Scientific, Inc., Waltham, MA, USA). The cells were incubated in a humidified atmosphere containing 5% CO_2_ at 37 °C (Thermo Fisher Scientific, Inc., Waltham, MA, USA).

### 4.2. Designing gRNAs

To validate the CRISPR activators, we selected *POU5F1* and *SOX2* as reference genes of human cells and designed gRNAs targeting their promoter regions. It has been reported that three to five gRNAs within 300–400 bp upstream region of transcription start site (TSS) are most effective to activate the transcription of *POU5F1*, *SOX2* and *IL1RN* [72]. So, we selected 400 bp region upstream to TSS for designing gRNAs by using Zhang Lab’s web tool (http://crispr.mit.edu/, Cambridge, MA, USA). For each gene, five promoter targeting gRNA sequences were selected based upon their score and relatively even distribution along the promoter region (Table S1). The strand orientation was not taken under consideration while selecting the gRNAs.

### 4.3. Construction and Confirmation of Array and Multiplexed CRISPR Plasmids

The Golden Gate cloning [128] was applied to construct multiplex plasmid by using the vectors pMA_spCas9_T# and pFUS_B10 (kindly gifted by Dr. Yonglun Luo, Department of Biomedicine, Aarhus University, Denmark). The 10 μM final concentration of oligonucleotides corresponding to each gRNA was annealed in ATP (1× final concentration; NEB Inc., Ipswich, MA, USA), T4 PNK buffer (1× final concentration; NEB) and T4 PNK enzyme (10 units; NEB). The annealing conditions for the thermocycler were adjusted to 95 °C for 5 min followed by a −5 °C/3 min ramp down to 65 °C; 63 °C for 3 min, followed by −3 °C/3 min ramp down to 27 °C; and a final incubation at 25 °C for 10 min. The PNK annealed oligonucleotides were diluted to a final concentration of 1 μM for cloning. In the first round of cloning, annealed oligonucleotides corresponding to each gRNA were cloned one by one into respective pMA_SpCas9_T# plasmids in a 20 μL reaction volume (30 ng annealed oligo, 50 ng of respective pMA_SpCas9_T#, 1 µL BbsI (NEB), 1 µL T4 DNA ligase (NEB), 2 µL 10 × T4 ligase buffer (NEB), nuclease-free water), followed by processing through thermal reaction (1 cycle: 37 °C for 5 min, 22 °C for 5 min; 15 cycles: 37 °C for 30 min; 75 °C for 15 min; and storage at 4 °C). The ligation mixture was transformed into the bacterial strain DH10B (Thermo Fisher Scientific, Inc., Waltham, MA, USA) which was further grown on 2XYT agar plate under ampicillin (Thermo Fisher Scientific, Inc., Waltham, MA, USA) selection (100 μg/mL). The grown colonies showing the right-sized amplicon after colony-PCR were confirmed by Sanger sequencing. PCR primers included the forward primer U6-F and the cloned oligo sequence as a reverse primer. The positive constructs were re-named as pMA_SpCas9_gRNA1 through pMA_SpCas9_gRNA10 depending upon the type of gRNA cloned into them. Next, a second round of cloning was conducted by digesting and ligating all eleven plasmids in the same 20 μL reaction mixture (50 ng of pFUS_B10 plasmid, 30 ng of all 10 pMA_SpCas9_gRNA1 to pMA_SpCas9_gRNA10 plasmids, 1 µL BsaI (Thermo Fisher Scientific, Inc., Waltham, MA, USA), 1 µL T4 DNA ligase (NEB), 2 µL 10 × T4 ligase buffer (NEB), nuclease-free water), followed by processing through thermal reaction (1 cycle: 5 min, 37 °C, 5 min 22 °C, 15 cycles: 30 min, 37 °C; 15 min, 75 °C; end: 4 °C). The blue-white screening under spectinomycin (Sigma-Aldrich, St. Louis, MO, USA) selection helped to pick the colonies for colony-PCR analysis. Primers for PCR include forward primer U6-F and reverse primer Scr-R. The positive construct was named pFUS_B_gRNA10 after confirmation through Sanger sequencing. The PCR mixtures was run at 2% agarose gel at 60 V for 1 h and visualized under a UV-illuminator. The CRISPR/dCas9 activator plasmids pCXLE-dCas9VP192-T2A-EGFP (cat #69536) and pLV hUbC-VP64 dCas9 VP64-T2A-GFP (cat #59791) were purchased from Addgene (Addgene, Watertown, MA, USA). The sequences of primers are mentioned in Table S2.

### 4.4. Transfection

HEK293T cells were seeded in a 6-well plate (BD Biosciences, San Jose, CA, USA) to reach 70–90% confluency before the transfection. All the transfection steps were performed with Lipofectamine 3000 including p3000 (Thermo Fisher Scientific, Inc., Waltham, MA, USA) by following the manufacturer’s protocol. The cells were co-transfected with a total of 2.5 µg of plasmids, including pFUS_B_gRNA10 and activator plasmid VP64-dCas9-VP64 or dCas9-VP192 at the same molar ratio. The cells without transfection served as a negative control in all the experiments. All the cells were incubated in humidified environment at 37 °C and 5% of CO_2_ for 24 h and the GFP signal was visualized by an inverted microscope (OLYMPUS IX53; Olympus Corporation, Tokyo, Japan).

### 4.5. Real Time RT-PCR (qRT-PCR)

HEK293T cells were seeded in a 6-well plate (BD Biosciences, San Jose, CA, USA) and transfected with relevant plasmids. Total RNA was isolated from the cells using the TRIzol^®^ Reagent (Invitrogen, Carlsbad, CA, USA). DNase treatment was done to eliminate genomic DNA contamination by means of the TURBO DNA-free™ Kit (Thermo Fisher Scientific Inc., Waltham, MA, USA). Total RNA concentration was measured on a micro UV-Vis fluorescence spectrophotometer (e-spect, Malcom, Japan), and 1 µg of RNA was used to synthesize cDNA in 40 μL reaction by using the iScriptTM cDNA Synthesis Kit (Bio-Rad Inc., Hercules, CA, USA). The mixtures were diluted five times with distilled water to obtain a final volume of 200 µL. The real time RT-PCR was carried out with the Light Cycler^®^ 480 SYBER Green I Master kit (Roche Diagnostics, Ottweiler, Germany). Gene quantification was conducted with a Rotor-Gene Q (Qiagen Sciences, Maryland, MD, USA) in a 20 μL reaction. Thermal cycling conditions were applied as follows: initiation for 10 min at 95 °C, followed by 40 cycles of 15 s at 95 °C, 30 s at 60 °C, and 15 s at 72 °C. The relative mRNA expression levels were calculated by the ∆∆CT method, where cyclophilin G (*PPIG*) served as an endogenous control and non-treated cells as a negative control. The sequences of primers used in real-time PCR are mentioned in Table S2. All of the PCR reactions were run in triplicate.

### 4.6. Western Blotting

The treated cells were centrifuged and washed with 1% PBS. Total protein was extracted using the M-PER mammalian protein extraction reagent (Thermo Fisher Scientific, Inc., Waltham, MA, USA). The bicinchoninic acid (BCA) assay kit (Sigma-Aldrich, St. Louis, MO, USA) was used to measure the protein. Mini-PROTEAN Tetra Cell and a mini trans-blot electrophoretic transfer cell system (Bio-Rad Laboratories, Hercules, CA, USA) were used for the Western blot analysis, including gel electrophoresis and transfer. A total of 30 μg of each protein sample was separated by SDS-PAGE and then transferred to a nitrocellulose membrane (Amersham Pharmacia Biotech, Buckinghamshire, UK). The membranes were blocked with 5% skim milk (BioRad Lab, Irvine, CA, USA) for 1 h, then were incubated overnight with 2000-fold-diluted primary antibodies specific to OCT-4A (POU5F1), CAS9, ERK, JNK (Cell Signaling Technology Inc., Danvers, MA, USA), SOX2 (Abcam, Cambridge, MA, USA), GFP, P65, or β-actin (Santa Cruz Biotechnology Inc., Dallas, TX, USA). Membranes were next incubated with anti-mouse or anti-rabbit horseradish peroxidase–conjugated secondary antibodies (Thermo Fisher Scientific Inc., Waltham, MA, USA) for 2 h at room temperature. The protein signals were developed by means of the SuperSignal West Pico ECL solution (Thermo Fisher Scientific Inc., Waltham, MA, USA), visualized on a ChemiDoc™ Touch Imaging System (Bio-Rad Laboratories), and processed through the EvolutionCapt software (Vilbert Lourmat, Collégien, France). The signal quantification for each relevant protein band was determined with the EvolutionCapt software (Vilbert Lourmat, Collégien, France). The bands were quantified by using the ImageJ software (National Institute of Health, Maryland, USA).

### 4.7. Immunocytochemical Staining

Cells were fixed with 4.0% formaldehyde (Sigma-Aldrich, St. Louis, MO, USA) for 5 min, followed by cell permeabilization with 0.2% Triton X-100 for 5 min. The blocking step involved incubation with 5% FBS (Thermo Scientific, Inc., Waltham, MA, USA) in PBS for 30 min and then 1 h with the 1000-fold-diluted anti-OCT-4A antibody (Cell Signaling Technology Inc., Danvers, MA, USA). The cells were then rinsed well with 1% PBS and incubated with 1000-fold-diluted secondary antibodies conjugated with Alexa Fluor 546 (Invitrogen, Carlsbad, CA, USA). Hoechst 33,258 solution (5 μM; Sigma-Aldrich, St. Louis, MO, USA) was added to stain the nuclei for 10 min. Each step required thorough washing with 1% PBS. The stained cells were visualised under the inverted microscope (Olympus IX53; Olympus Corporation, Tokyo, Japan) and processed through IMT iSolution Auto Plus software (IMT i-solution Inc., Vancouver, BC, Canada).

### 4.8. Off-Target Prediction

The Cas-OFFinder tool (http://www.rgenome.net/cas-offinder/, Cambridge, MA, USA) was used to predict the off-targets for all 10 gRNAs used in our study. The off-targets were searched for *Streptococcus pyogenes* Cas9 enzyme (protospacer adjacent motif sequence: NGG) with 3 mismatches in GRCh38/hg38 target genome of *Homo sapiens*. The distance of off-targets from the transcription start site (TSS) of nearby genes and the reference sequence accession number (RefSeq IDs) of that gene were obtained using the Homer software (http://homer.ucsd.edu/homer/ngs/annotation.html, San Diego, CA, USA). The genes with off-targets located between 0 and −400 bp of TSS were selected to design primers using the primer designing tool of the National Center for Biotechnology Information. The complete list of off-targets is mentioned in Data File S1 (Supplementary Materials), while the primers used for the qRT-PCR are mentioned in Table S2.

### 4.9. Statistical Analysis

All the in vitro data analyses were performed using Tukey’s method one-way ANOVA. All the data are presented as means of three independent experiments where bars represent ± SEMs. *p* value < 0.05 was considered statistically significant.

## 5. Conclusions

Taken together, these data support the usage of CRISPR/dCas9 activator systems for the specific transcriptional upregulation of target genes. They also highlight that increasing the number of transcription activator units could increase the extent of transcriptional activation. For instance, the selection of an appropriate activator system for *POU5F1* and *SOX2* can support the efficient generation of iPSCs. Moreover, the optimally efficient activation of endogenous genes could be helpful in medicine, agriculture, and biotechnology.

## Data Availability

The data presented in this study are available in article and supplementary material.

## Supplementary Materials

The following are available online at https://www.mdpi.com/1422-0067/22/1/397/s1: Figure S1: construction of multiplex plasmid with cloned *POU5F1* and *SOX2* targeting gRNAs; Figure S2: effect of CRISPR activators on some random cellular proteins; Figure S3: evaluation of off-target effect by CRISPR activator system. Table S1: selected gRNAs specific to *POU5F1* and *SOX2* genes’ promoters by Zhang Lab’s web tool; Table S2: primers for colony PCT, qRT-PCR, and off-target analysis. Data File S1: complete list of the predicted off-targets.
