# Supplementary material for: Functional Comparison between VP64-dCas9-VP64 and dCas9-VP192 CRISPR Activators in Human Embryonic Kidney Cells
Source: Int J Mol Sci. 2021 Jan 1;22(1):397. doi: 10.3390/ijms22010397 (PMC7795359; doi:10.3390/ijms22010397)
Supplement: Supplementary file 1 [file ijms-22-00397-s001.zip › ijms-1050984 supplementary/Supplemantary Figures and Tables.pdf]

International Journal of *Molecular Sciences*

MDPI

Article

Functional comparison between VP64-dCas9-VP64 and dCas9-VP192 CRISPR activators in human embryonic kidney cells

Nasir Javaid[1†], Thuong L.H. Pham[1†], Sangdun Choi[1,2*]

[1] Department of Molecular Science and Technology, Ajou University, Suwon, 16499, Korea
[2] S&K Therapeutics, Woncheon Hall 135, Ajou University, Suwon, 16499, Korea
[†] These authors contributed equally
[*] Correspondence: sangdunchoi@ajou.ac.kr; Tel.: +82 31-219-2600

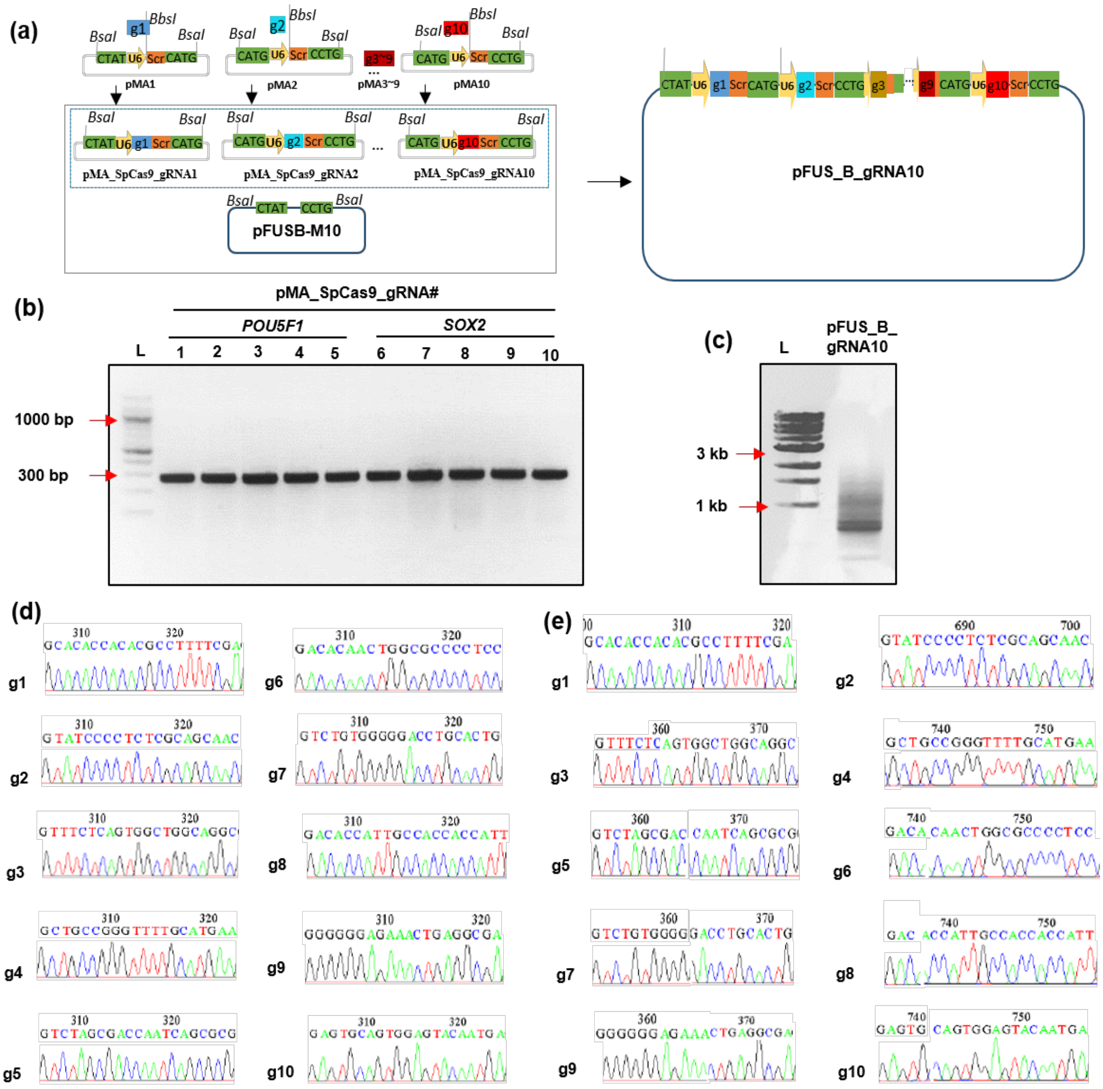

Figure S1. Construction of multiplex plasmid with cloned *POU5F1* and *SOX2* targeting gRNAs. **(a)** The gRNAs (g1~g10) were firstly cloned to array plasmids (pMA1~pMA10) by *BbsI* restriction enzyme, then the positive clones (pMA_SpCas9_gRNA1~pMA_SpCas9_gRNA1) were assembled into the plasmid backbone pFUSB-M10 by *BsaI* to create a multiplex plasmid pFUS_B_gRNA10 **(b)** The positive clones of array plasmids, pMA_SpCas9_gRNA1 through pMA_SpCas9_gRNA10, were confirmed with colony PCR by using forward primer U6-F and the respective cloned oligo sequence as a reverse primer (Table S2). L =100bp **(c)** Validation of multiplex plasmid, pFUS_B_gRNA10, was performed with colony PCR by using forward primer U6-F and reverse primer Scr-R which bind to each expression cassette. L =1kb. The PCR mixtures were run on 2 % agarose gel at 60 V for 1 hr and visualized under UV-illuminator. **(d, e)** The confirmation of each array plasmid, pMA_SpCas9_gRNA1 through pMA_SpCas9_gRNA10, **(d)** and multiplex plasmid, pFUS_B_gRNA10, **(e)** were done by sanger sequencing. gRNA, guide RNA. L = DNA ladder.

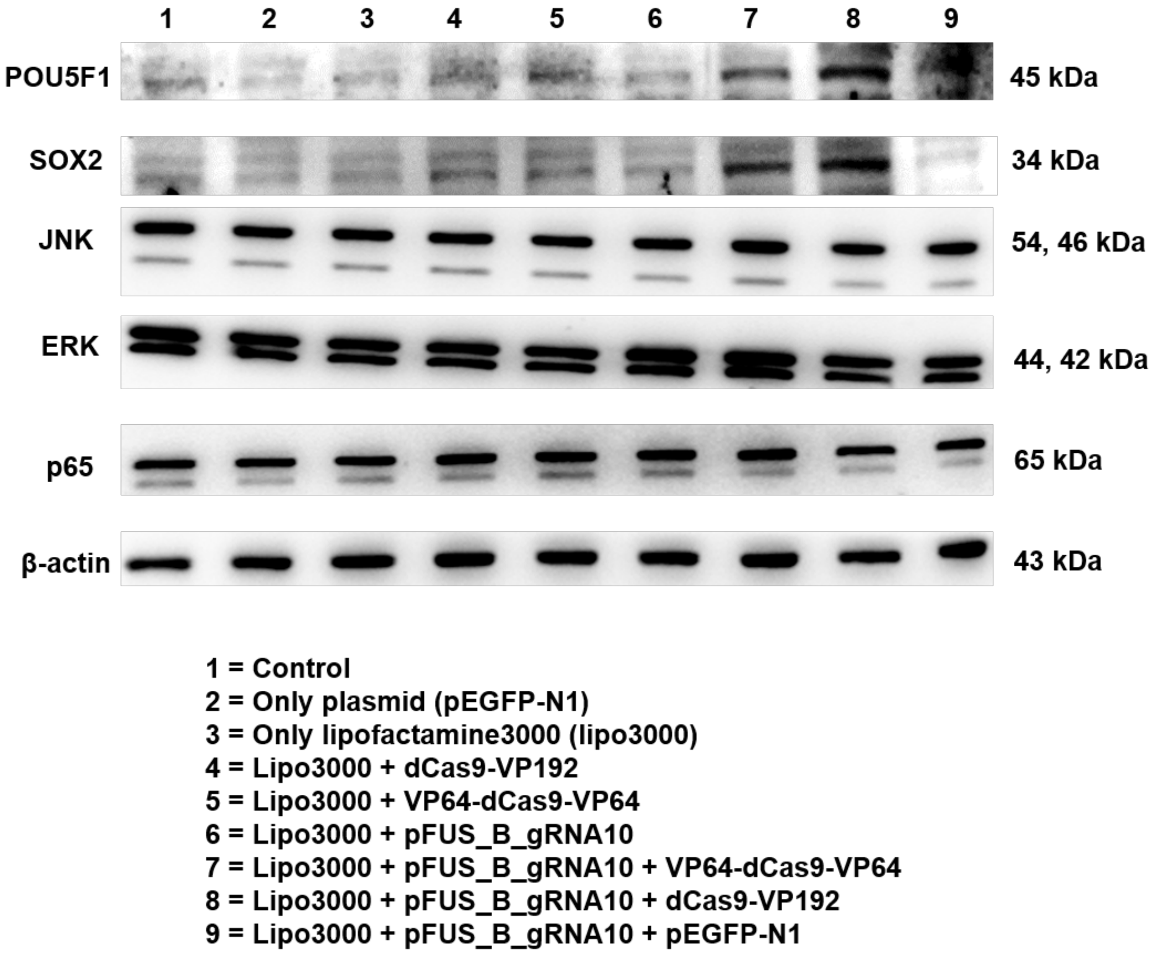

Figure S2. Effect of CRISPR activators on some random cellular proteins. HEK293T cells were transfected with only plasmid, only lipofactamine3000 and/or together with multiple combinations of plasmids (as labelled) for 72 hrs. Afterwards, total cell proteins were isolated; separated by electrophoresis; transferred to nitrocellulose membrane; and immunoblotted with respective antibodies. The β-actin served as an internal and loading control.

(a)

crRNA sequence	Off-target sequence	Locus	Distance to TSS (bp)	Gene name
GACACAACTGGCGCCCCTCCNGG	GACACAACcGGCGgCtCTCCGGG	Chr1: -202861559	-184	ACTG1P25
GGGGGGAGAAACTGAGGCGANGG	GGGtGGgGAAACTGAGGCGgAGG	Chr2: +113890344	-243	ACTR3
TCTAGCGACCAATCAGCGCGNGG	TCgcGCGgCCAATCAGCGCGCGG	Chr4: -1721455	-31	TACC3
TTTCTCAGTGGCTGGCAGGCNGG	TTTCgCAGTGGaaGGCAGGCAGG	Chr5: -150289890	-61	CAMK2A
TCTAGCGACCAATCAGCGCGNGG	gCcAGCGgCCAATCAGCGCGCGG	Chr7: +16753693	-51	TSPAN13
TTTCTCAGTGGCTGGCAGGCNGG	cTgCTCAGTGGCTGGCgGGCGGG	Chr9: +136728311	-138	SNHG7
GGGGGGAGAAACTGAGGCGANGG	GGGcGGAGAAACTGAGGCctCGG	Chr19: -4343436	-80	MPND
TCTGTGGGGGACCTGCACTGNGG	TCaGTGaGGGAtCTGCACTGTGG	Chr19: +8609098	-239	ADAMTS10
GCTGCCGGGTTTTGCATGAANGG	GCTGCCGGGTcacGCATGAAGGG	Chr22: +22922849	-280	LOC105372947

(b)

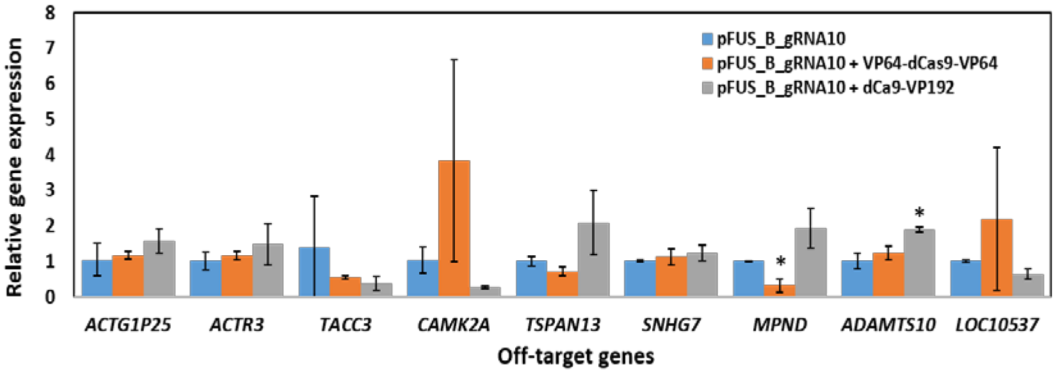

Figure S3. Evaluation of off-target effect by CRISPR activator system. **(a)** The off-target sites located between 0 to -400 bp distance from transcription start site (TSS) with three mismatches were selected by using off-target prediction tool. The mismatches are indicated with small red font. **(b)** After 72 hrs of transfection, total RNA was isolated and proceeded for gene expression analysis of off-targets via the SYBR Green detection method. CyclophilinG gene served as an endogenous control. The primers specific to each gene are enlisted in Table S2. The represented values are the average of two independent experiments where bars represent ± SEM (* $p < 0.05$, ** $p < 0.01$, *** $p < 0.001$) according to Tukey's method one-way ANOVA.

Table S1. Selected gRNAs specific to *POU5F1* and *SOX2* genes' promoters by Zhang Lab's web tool.

Target	Name	Target sequence	gRNA Oligo sequences
SOX2	g1	CACACCACACGCCTTTTCGA	F 5′-CACCGCACACCACACGCCTTTTCGA
			R 5′-AAACTCGAAAAGGCGTGTGGTGTGC
	g2	GTATCCCCTCTCGCAGCAAC	F 5′-CACCGTATCCCCTCTCGCAGCAAC
			R 5′-AAACGTTGCTGCGAGAGGGGATAC
	g3	TTTCTCAGTGGCTGGCAGGC	F 5′-CACCGTTTCTCAGTGGCTGGCAGGC
			R 5′-AAACGCCTGCCAGCCACTGAGAAAC
	g4	GCTGCCGGGTTTTGCATGAA	F 5′-CACCGCTGCCGGGTTTTGCATGAA
			R 5′-AAACTTCATGCAAAACCCGGCAGC
	g5	TCTAGCGACCAATCAGCGCG	F 5′-CACCGTCTAGCGACCAATCAGCGCG
			R 5′-AAACCGCGCTGATTGGTCGCTAGAC
POU5F1	g6	GACACAACTGGCGCCCCTCC	F 5′-CACCGACACAACTGGCGCCCCTCC
			R 5′-AAACGGAGGGGCGCCAGTTGTGTC
	g7	TCTGTGGGGGACCTGCACTG	F 5′-CACCGTCTGTGGGGGACCTGCACTG
			R 5′-AAACCAGTGCAGGTCCCCCACAGAC
	g8	ACACCATTGCCACCACCATT	F 5′-CACCGACACCATTGCCACCACCATT
			R 5′-AAACAATGGTGGTGGCAATGGTGTC
	g9	GGGGGGAGAAACTGAGGCGA	F 5′-CACCGGGGGGAGAAACTGAGGCGA
			R 5′-AAACTCGCCTCAGTTTCTCCCCCC
	g10	AGTGCAGTGGAGTACAATGA	F 5′-CACCGAGTGCAGTGGAGTACAATGA
			R 5′-AAACTCATTGTACTCCACTGCACTC

Table S2. Primers for colony PCT, qRT-PCR, and off-target analysis

**Experiment**	**Target**	**Primer**	**5′-Sequenceg-3′**
Colony PCR	*U6*	Forward	GAGGGCCTATTTCCCATG
	SCR	Reverse	TAACTTGCTATTTCTAGCTC
qRT-PCR	*POU5F1*	Forward	TTGGGCTCGAGAAGGATGTG
		Reverse	TCCTCTCGTTGTGCATAGTCG
	SOX2	Forward	GCCCTGCAGTACAACTCCAT
		Reverse	TGCCCTGCTGCGAGTAGGA
	PPIG	Forward	TCTTGTCAATGGCCAACAGAG
		Reverse	GCCCATCTAAATGAGGAGTTG
Off-targets	*ACTG1P25*	Forward	CCTCGGCTGATCACAATGGA
		Reverse	TCGGGGAACTATACCCAGCA
	ACTR3	Forward	TTTTTGCCAGTCGGTTTGGG
		Reverse	ACCAGGAGCGCTTCTCTTTTC
	TACC3	Forward	CATCCTGGAGCTCGCCATAC
		Reverse	CACATCAGATCCGCAGGGAA
	CAMK2A	Forward	TTCTCTGTTTGCACTCGGCA
		Reverse	CAGGTGAGGCTTGGGACTG
	TSPAN13	Forward	TCCGCCGGAGTCGAATTTAC
		Reverse	GTTCTTGGAACACGCGAAGC
	SNHG7	Forward	CAGTTCTCGAGCGCCTCAC
		Reverse	CAGCAGCCCAGTCCCCTA
	MPND	Forward	TCCTCCCGAGATGCTGCT
		Reverse	TTGTCGAGGTAGGTGTGCTC
	ADAMTS10	Forward	CAAAGCACAGAAGGGGGTGT
		Reverse	TGTCCTGGTCCCCCTAAGAC
	LOC105372947	Forward	TCACCTATAGACCCCCACCG
		Reverse	TCAGACAGACACCCCCAAGA
